# Exploring the Potential of Primary ECT Modulation: A Transformative Approach in Schizophrenia Treatment

**DOI:** 10.1192/bjo.2024.667

**Published:** 2024-08-01

**Authors:** Ahmad Rehan Khan, Oyku Inanc, Sukhmani Kaur Sadana, Ania Fida

**Affiliations:** 1 Carilion New River Valley Medical Center, Christiansburg, Virginia, USA; 2 Gulhane Training and Research Hospital, Ankara, Turkey; 3 West Tennessee Healthcare, Tennessee, USA; 4 Medical College of Wisconsin, Wisconsin, USA

## Abstract

**Aims:**

Electroconvulsive therapy (ECT) stands as a crucial intervention for severe and treatment-resistant schizophrenia. Despite being recognized as the most effective acute treatment for severe mood and psychotic disorders, its controversial nature persists due to misconceptions and a lack of familiarity among healthcare professionals regarding modern ECT techniques. This case explores the effectiveness of maintenance ECT in preventing relapse among individuals with schizophrenia, a dimension with scarce existing data.

**Methods:**

A 28-year-old unemployed Caucasian male with treatment-resistant schizophrenia underwent multiple trials of atypical, typical, and depot antipsychotics, yielding no significant improvement in the Positive and Negative Syndrome Scale (PANSS) score. Two attempts with clozapine were hindered by neutropenia. With a baseline PANSS symptom score of 110, the patient struggled with severe auditory and visual hallucinations, preventing coherent conversations. Following 26 sessions of bilateral ECT, the PANSS scale score decreased to 65, prompting transfer to a Transitional Living Facility. After an additional 14 sessions, the patient exhibited significant symptomatic improvement, leading to discharge. The PANSS scale score, after 40 sessions, reached 50. Monthly bilateral ECT sessions and one antipsychotic medication now maintain the patient's reasonably functional lifestyle, encompassing employment, social outings, and assistance in farming with his father. ECT proved highly successful in alleviating both positive and negative symptoms, transforming the patient from severe conversational impairment to independent living and employment.

**Results:**

Empirical data validates clozapine's efficacy for treatment-resistant schizophrenia, yet its clinical use is limited by the substantial risks of agranulocytosis and neutropenia, relegating it to a third-line option. Neutropenia's onset in our case during clozapine trials prompted a therapeutic shift to electroconvulsive therapy (ECT). Aligned with American Psychiatric Association guidelines, our case underscored ECT's superior efficacy compared with traditional antipsychotics. Acknowledging a 40% non-response rate to clozapine across diverse studies emphasizes ECT's significance as a viable alternative. Despite challenges, contemporary ECT methods promise to overcome traditional constraints, reduce stigma, and improve treatment accessibility.

**Conclusion:**

This case underscores the potential benefits of ECT as a valuable treatment modality for individuals with treatment-resistant schizophrenia, effectively managing both positive and negative symptoms and significantly improving daily functioning. The success observed in this case suggests that monthly bilateral ECT and one antipsychotic medication can play a crucial role in enhancing the quality of life for patients with treatment-resistant schizophrenia.

